# Gay father surrogacy families: relationships with surrogates and egg donors and parental disclosure of children's origins

**DOI:** 10.1016/j.fertnstert.2016.08.013

**Published:** 2016-11

**Authors:** Lucy Blake, Nicola Carone, Jenna Slutsky, Elizabeth Raffanello, Anke A. Ehrhardt, Susan Golombok

**Affiliations:** a Centre for Family Research, University of Cambridge, Cambridge, United Kingdom; b Department of Developmental and Social Psychology, Sapienza University of Rome, Rome, Italy; c Division of Gender, Sexuality, and Health, New York Psychiatric Institute, Columbia University, New York, New York

**Keywords:** Children, disclosure, egg donor, gay father, surrogacy

## Abstract

**Objective:**

To study the nature and quality of relationships between gay father families and their surrogates and egg donors and parental disclosure of children's origins.

**Design:**

Cross-sectional study.

**Setting:**

Family homes.

**Patient(s):**

Parents in 40 gay father families with 3–9-year-old children born through surrogacy.

**Intervention(s):**

Administration of a semistructured interview.

**Main Outcome Measure(s):**

Relationships between parents, children, surrogates, and egg donors and parental disclosure of children's origins were examined using a semistructured interview.

**Result(s):**

The majority of fathers were content with the level of contact they had with the surrogate, with those who were discontent wanting more contact. Fathers were more likely to maintain relationships with surrogates than egg donors, and almost all families had started the process of talking to their children about their origins, with the level of detail and children's understanding increasing with the age of the child.

**Conclusion(s):**

In gay father surrogacy families with young children, relationships between parents, children, surrogates, and egg donors are generally positive.

**Discuss:** You can discuss this article with its authors and with other ASRM members at **https://www.fertstertdialog.com/users/16110-fertility-and-sterility/posts/11529-gay-father-surrogacy-families-relationships-with-surrogates-and-egg-donors-and-parental-disclosure-of-children-s-origins**

In the United States it is has been estimated that between 2 and 3.7 million children have a lesbian, gay, bisexual, or transgender parent, with approximately 200,000 being raised by same-sex couples (1). Given recent changes in marriage equality in the United States (2) and physicians’ ethical obligation to treat all persons equally regardless of sexual orientation (3), the number of gay fathers creating families through assisted reproductive technologies is likely to rise.

Gay men may choose to become parents via surrogacy, a process in which a woman bears a child for the intended parent(s). This can be a relatively low-technology procedure in which conception occurs using the sperm of one of the intended fathers and the egg of the surrogate who carries the child to term (referred to as genetic surrogacy). However, the most common type of surrogacy in the United States is gestational surrogacy (4), a high-technology procedure in which an embryo is created using the sperm of one of the intended fathers and the egg of a donor and transferred to the surrogate. The surrogate who carries the pregnancy to term and gives birth has no genetic connection to the child.

Concerns have been expressed regarding the relationship between families created through surrogacy and the surrogate over time (5). Although contact with the surrogate may be beneficial in helping children understand their origins, there have been fears that ongoing contact with the surrogate may undermine the relationship between the parents and the child. These concerns have typically been raised in relation to heterosexual parents, specifically mothers, as opposed to fathers in gay father families.

Studies of surrogacy families conducted in the United Kingdom, where commercial surrogacy is illegal, have found that heterosexual parents can and do form positive relationships with surrogates during pregnancy, which typically continue as the child grows up 6, 7. However, the amount of contact between children and their surrogate has been found to lessen over time, particularly in the case of previously unknown genetic surrogates (6).

A small body of research has examined the relationship between gay fathers and their surrogates in Spain, Italy, and the United States 8, 9, 10 both during and immediately after the birth of the child. Relationships between fathers and surrogates have generally been found to be positive, with contact being maintained through occasional emails and/or the exchange of postcards and photographs at birthdays and holidays. When contact between gay fathers and surrogates has been found to cease entirely, this has occurred in the Indian context (11), in which socioeconomic and language barriers, as well as agency policies, do not encourage or facilitate contact between parties (12).

In gestational surrogacy arrangements, parents may select an egg donor with whom they can have contact in the future (an open-identity donor) or a donor with whom they will have no contact (an anonymous donor), although the possibility of achieving anonymity is increasingly in doubt (13). A relationship between the child and the egg donor may be viewed by intended parents as threatening, given that genetic relatedness is often given primacy in family relationships (14). Even where there is no relationship between the child and the donor or surrogate, it has been argued that these “birth others” (15) may have a place in the child's family tree (16). Despite the fact that 18,400 infants were born in the United States through gestational surrogacy between 1999 and 2013 (4), the nature of the relationship between children in these families and their egg donor is unknown.

Gay fathers who started their families using surrogacy need to explain their path to parenthood to their children. In surrogacy families headed by heterosexual couples, almost all parents are open with their children about their use of a surrogate 6, 17. This openness is unsurprising, given that the parents have to explain the arrival of the baby to family and friends in the absence of a pregnancy. A high level of openness is likewise to be expected in gay father families given the absence of a partner of the opposite-sex with whom to procreate. However, the specific aspects of the surrogacy process that gay fathers choose to disclose to their children, and at what age they choose to do, have not been studied. When children in the United Kingdom longitudinal study of surrogacy families headed by heterosexual couples were 10 years old (6), 58% of parents with a genetic surrogate had told their children about the surrogate but had not mentioned the fact that the surrogate's egg had been used in their conception. This partial disclosure suggests that the use of a surrogate in the gestation and birth of a child may be easier to disclose to young children than is the use of donor eggs. In addition to explaining the role of the surrogate and the egg donor, gay couples may or may not tell their children which father has a genetic connection to the child.

Relationships between fathers and surrogates have been found to be positive both during and immediately after the birth of the child. However, little is known about how relationships with both surrogates and egg donors change over time as children develop an increasingly sophisticated understanding of their birth, or how and when fathers talk about surrogacy with their children. Therefore the present study examined three questions in a sample of gay father families with 3–9-year-old children born through surrogacy: [1] Do gay fathers and their children have contact with their surrogates and egg donors? [2] What kind of relationships do gay fathers and their children have with their surrogates and egg donors? [3] What have parents explained to their children about their surrogacy origins?

## Materials and methods

### Sample

Forty gay father families created through surrogacy participated in the study, all of whom resided in the United States. The inclusion criteria for participation were that the target child was aged between 3 and 9 years and the parents had been a couple since the time of the child's birth.

Families were recruited through the use of multiple strategies. First, surrogacy agencies that specialized in working with gay men sent information about the study to the fathers in their mailing list (n = 18, 45%); second, participants passed on information about the study to their friends, colleagues, or acquaintances who fit the study criteria and/or disseminated information about the study through social media (n = 7, 17.5%); and third, families were recruited at events at which gay fathers were in attendance (n = 15, 37.5%).

There were 24 boys (60%) and 16 girls (40%) in the sample, with an average age of 5 years 8 months (SD 2.2 years). The mean age of the fathers was 47.29 years (SD 6.20 years). The mean annual family income was $370,000 (SD $168,264). Most fathers were white (n = 67, 84%), with the remaining fathers identifying as Latino/Hispanic (n = 7, 9%), Asian (n = 1, 1%), or “other” (n = 5, 6%). Ninety-eight percent of fathers had a bachelor's or higher degree. Most families lived in the Northeast (67.5%; New York City = 24, Massachusetts = 3), with the remaining families living in the South (7.5%; Florida = 1, Virginia = 1, Texas = 1), the West (22.5%; California = 7, Oregon = 1, Washington = 1), and the Midwest (2.5%; Minnesota = 1).

### Procedure

Ethical approval was granted by the New York State Psychiatric Institute Institutional Review Board and the University of Cambridge Psychology Research Ethics Committee. Fathers were given an information sheet and an opportunity to ask any questions before signing a consent form and beginning participation. The majority of families were visited in their homes by a research psychologist trained in the study techniques (n = 26, 65%). Because of geographic distance from the researcher, data were collected from the remaining families over Skype (n = 14, 35%), which has been recognized as both commonplace in social science research and a viable methodologic approach (18).

### Measures

The primary aim of the study was to investigate parent–child relationships and child adjustment in gay father families formed through surrogacy. Both fathers were interviewed separately (19), and interviews were digitally recorded. A section of the interview focused on fathers' experiences of surrogacy, using questions adapted from the United Kingdom longitudinal study of heterosexual families formed through surrogacy (7). Fathers were asked about the relationship they and their children had with the surrogate and the egg donor (including the frequency and method of contact and the general quality of the relationship). Fathers were also asked about the process of telling their children about their origins, focusing on the frequency and content of discussions they had had with their children about the surrogacy process, the use of an egg donor (if applicable), and whose sperm was used in the child's conception. Interview data have been presented from the father who identified as being the most involved with the child/children on a day-to-day basis (labeled for this analysis as parent A). This distinction was straightforward in most families (n = 27, 67.5%), and in the remaining families where time with children was fairly even between fathers (n = 13, 32.5%) the label of parent A was assigned randomly.

### Analytic Approach

The section of the interview on fathers' experiences of surrogacy was transcribed and analyzed by two of the authors (L.B. and N.C.) using a content analysis approach (20). The analysis was guided by the principles of qualitative description, which aims to report participants' motivations and experiences in as close a way as possible to their own interpretation 21, 22. Because the interviews were semistructured, the data of interest were dispersed throughout the transcript. Therefore, data were organized into excel sheets (e.g., all quotes throughout the interview pertaining to “quality of relationship with surrogate” were copied into one cell). A coding manual was created that described the information in each cell succinctly. The interviews were then rated in accordance with the coding manual, and frequency counts were calculated. Where appropriate, comparisons between the nature of fathers’ relationships with surrogates and egg donors were conducted using χ^2^ tests, and the strength of the association between variables was assessed through correlation.

One-third of the transcripts were recoded by a second researcher to calculate interrater reliability. Percentage agreement was 90% or above for each variable, categorized as follows: [1] Path to parenthood: Location (United States, India); surrogacy arrangement (genetic, gestational); relationship to surrogate (unknown, sister, friend); relationship to egg donor (unknown, sister, friend), meetings with unknown egg donor (yes, no, telephone); egg donor status (open identity, anonymous). [2] Contact with surrogate and egg donor: Met since child born (yes, no); met in past year (yes, no); number of meetings in past year (1–2, 3 or more); methods of contact: phone, email, Skype, text message, Facebook, cards/gifts/flowers (yes, no); met with fathers' family (yes, no). [3] Quality of relationship with surrogate and egg donor: Happy with level of contact (content, neutral, discontent, no contact); quality of relationship with surrogate/egg donor (positive, neutral, negative, no relationship); quality of child's relationship with surrogate/egg donor (close, neutral, distant, nonexistent). [4] The disclosure process: Started the process of disclosure (yes, no); stages of disclosure: two dads need help to have a baby (yes, no), babies carried in women's tummies (yes, no), specific reference to surrogate (yes, no), disclosure of donated egg (yes, no), disclosure of whose sperm was used (yes, no); use of children's books (yes, no); photos of the surrogate (yes, no); homemade books/photo albums/videos (yes, no); children's understanding (none, some understanding, understands all, don't know).

## Results

### Path to Parenthood

The majority of surrogacy arrangements were carried out in the United States (n = 38, 95%), with two (5%) conducted in India. Most surrogacy arrangements were gestational (n = 36, 90%), with four couples (10%) conceiving via genetic surrogacy.

In families formed through gestational surrogacy, most surrogates were previously unknown to the couple (n = 35, 97%), and one surrogate (3%) was a friend. In genetic surrogacy families, three surrogates (75%) were previously unknown to the couple, and one was a sister of the nongenetic father (25%).

In gestational arrangements, most parents had used an unknown egg donor (n = 34, 94%), with one couple using a friend (3%) and one using a sister (3%). Only four fathers described donors whom they had never met with, seen, or spoken with, and with whom they had very little chance of making contact or meeting in the future (12%). All other donors were categorized as open-identity (n = 30, 88%). Of the 30 fathers who conceived using an open-identity egg donor, 18 fathers (60%) had met the donor, and 2 fathers (7%) had spoken to her on the phone. Of the four fathers who conceived using an anonymous egg donor, none had met or spoken to her, but one father thought that he had seen her at the clinic by chance.

### Contact with Surrogate and Egg Donor

As shown in Table 1, after the birth of the child a greater percentage of fathers had met with the surrogate (n = 33, 83%) than had met with the egg donor (n = 9, 25%) (χ^2^ = 25.34, *P*<.01). Similarly, fathers were more likely to have met with the surrogate in the past year (n = 21, 53%) compared with the egg donor (n = 2, 6%) (χ^2^ = 19.79, *P*<.01). In the two families in which fathers had met with the egg donor in the past year, the egg donor was previously known to the couple (one sister, one friend).

At the time of the interview, fathers were more likely to maintain a relationship with the surrogate (n = 34, 85%) than with the egg donor (n = 11, 31%) (χ^2^ = 23.25, *P<*.01). Of the 11 parents who maintained contact with egg donors, 9 were open-identity egg donors, and 2 were known (one friend, one sister). The most popular methods for maintaining contact were Facebook and e-mail, and the least common methods were phone calls and Skype.

Surrogates were more likely to have met the fathers' extended family (n = 27, 68%) than were egg donors (n = 6, 17%) (χ^2^ = 19.93, P<.01). Surrogates had met the fathers' brothers or sisters (n = 18, 66%) and attended weddings (n = 4, 15%) and/or baby showers (n = 5, 19%). Of the six egg donors who had met with the fathers’ parents and siblings, four were open-identity egg donors, and two were known (one friend, one sister).

### Quality of Relationship with Surrogate and Egg Donor

Of the 34 fathers (85%) who were in contact with the surrogate, the majority (n = 32, 78%) were content or neutral about their level of contact, with those fathers who were discontent (n = 2, 6%) wanting more contact with her (Table 1). Of those who maintained contact with the egg donor (n = 11, 31%), most were content or neutral about their level of contact (n = 7, 63%), and four fathers (36%) were discontent and wished to have more contact with her. Fathers were more likely to be discontent with the level of contact they had with the egg donor compared with the surrogate (χ^2^ = 4.17, *P<*.05).

For those fathers who had an ongoing relationship with the surrogate (n = 34, 85%), most had a positive (n = 18, 53%) or neutral (n = 15, 44%) relationship with her. Likewise, of those fathers who had an ongoing relationship with the egg donor (n = 11, 31%), all were positive (n = 6, 55%) or neutral (n = 5, 45%). There was no difference between the quality of fathers’ relationships with surrogates and egg donors (positive/neutral vs. negative).

Most of the children had met with the surrogate (n = 33, 83%). In these families, the relationship between the child and the surrogate was mostly categorized as nonexistent (n = 7, 21%), distant (n = 8, 24%), or neutral (n = 10, 31%). In a minority of families, the relationship between the child and the surrogate was close (n = 7, 23%). The quality of the relationship between the child and the surrogate was significantly correlated with the frequency with which the surrogate had visited the family in the past year (*r* = 0.71, *P*<.01), showing that greater contact with the surrogate was associated with a closer relationship with the child.

Of the nine families (25%) in which fathers had met with the egg donor since the birth of the child, the relationship between the egg donor and the child was most often categorized as nonexistent (n = 5, 56%) or distant (n = 1, 11%). In two families (22%), the child had a close relationship with the egg donor, one of whom was a relative and the other a family friend. There was no difference between the quality of the child's relationship with the surrogate and the egg donor (close vs. not close).

### The Disclosure Process

The majority of fathers (n = 33, 83%) had started to talk to their children about their origins (Table 2). Of those who had begun to disclose, three-quarters (n = 25, 76%) had discussed the fact that two men need help in creating a family because babies are carried by women, and more than two-thirds had made specific reference to the surrogate who helped them (n = 23, 70%). In contrast, only one-third of fathers who had used gestational surrogacy (n = 12, 33%) had mentioned the use of a donated egg. Few fathers (n = 7, 21%) had begun to disclose which partner's sperm had been used in the child's conception. The older the child, the higher the stage of disclosure that parents had reached (*r* = 0.63, *P*<.01).

Half of the fathers felt that their children understood some of what had been explained to them (n = 20; 50%), and one-fifth (n = 8; 20%) thought that their children had a good understanding of their origins. Parents' reports of their children's understanding of their origins were significantly correlated with the age of the child (*r* = 0.42; *P*<.05), with greater understanding associated with the older age of the child.

## Discussion

Three main findings emerged from this study regarding the relationship between gay father families, surrogates, and egg donors when the children were in early and middle childhood. First, most fathers were content with the level of contact they had with their surrogate, with those who were discontent wishing to have greater contact. Second, fathers were more likely to actively maintain a relationship with the surrogate than the egg donor. Third, most fathers had started the process of explaining their path to parenthood to their children, with the level of details and children's understanding increasing with the age of the child.

The quality of the relationship between gay fathers and their surrogate was generally found to be positive, with most surrogates having met the child and other members of the fathers’ families during occasions such as baby showers and weddings. These findings are similar to those from surrogacy families headed by heterosexual couples 6, 7, 23, 24.

The classification of the egg donor as anonymous or open-identity was problematic, with some fathers having exchanged contact information with, and met, their “anonymous” egg donor. Studies of egg donation families headed by heterosexual couples likewise have found that parents know the name of their “anonymous” donor, in addition to having a description of her physical characteristics and medical history (25). In the present study a donor was labeled as anonymous only when there was little chance of the fathers being able to contact her in the future. Regardless of whether donors are categorized as anonymous, open-identity, or known, the reality of the situation over time may be different from the one that parents had imagined at the start of the process.

In line with previous research on gay father surrogacy families, fathers were more likely to maintain a relationship with the surrogate than with the egg donor 8, 9, 26. This discrepancy may be explained by inherent differences in the procedures: intended parents and surrogates have the opportunity to develop a relationship over many months, whereas egg retrieval is brief. Fathers may also choose a surrogate with characteristics that will increase the chance of a successful pregnancy as well as contact in the future, and an egg donor whose fertility is optimal 8, 9, 26, 27, 28. Although the egg donor is, for the most part, invisible in gay father surrogacy families when children are in their preschool or early school years, some fathers had deliberately chosen an egg donor with whom there would be the possibility of contact, so that their child's questions might be answered in the future. It is possible, therefore, that contact with the egg donor may occur or become more frequent when the child has a better understanding of, and curiosity about, their origins.

This study is the first to examine the nature of the relationship between the children in gay father families and the surrogate and egg donor who participated in their conception. For most children, it seemed that the relationship with their surrogate was neutral or distant. Relationships between children and egg donors were less common, but in a similar vein, neutral in nature. Concerns that these “birth others” may be a threat to the strength and unity of the family therefore seem to be unfounded, although future research is required to explore how these relationships evolve over time.

As for the process of disclosure, most fathers had started this process (83%), although few had disclosed the use of an egg donor or the identity of the genetic father. These findings echo those of surrogacy families with heterosexual parents, in which “partial disclosure” (disclosure of surrogacy with no reference to the egg donor) was common (6). The older the age of the child, the more layers of the disclosure story they had been told, but the precise age at which fathers will disclose the use of an egg donor and/or the identity of the genetic father remains to be seen.

A limitation of the study was the use of a volunteer sample, because it is possible that those fathers who have had a particularly positive experience may be more likely to participate in research. A variety of recruitment procedures were used to access as diverse a sample as possible, although gay father surrogacy families will most likely be unique in terms of income, given the high cost of pursuing surrogacy as a path to parenthood in the United States (5). As the number of gay father surrogacy families grows over time, future researchers can optimize recruitment strategies to increase the likelihood of obtaining a representative sample. It is also important that future research explores the quality of relationships with surrogates and egg donors from the perspective of the child, whose voices in these families have not yet been heard.

In conclusion, the findings of this study suggest that the concerns raised regarding surrogates and egg donors interfering in family life are unfounded. Although surrogacy arrangements have been expected to be more positive when entered into on altruistic grounds (29), the findings of the present study suggest that the commercial basis of the US system may also be conducive to positive and successful surrogacy arrangements. The convenience nature of this sample must be taken into account, because fathers who have had a particularly positive experience may be more likely to participate in research. However, the findings are consistent with the literature on heterosexual parent families created by surrogacy 5, 6, 7, 23, 24, showing positive relationships between parents, children, and surrogates.

## Figures and Tables

**Table 1:** Contact and quality of relationship with surrogate and egg donor.

Variable	Surrogate (n = 40), n (%)	Egg donor (n = 36), n (%)	χ^2^	Illustrative quotes (SU = surrogate, ED = egg donor)
Met since child born			25.34, *P*<.01	“We went to visit once with [child] shortly after he was born and then again after [other child] was born. We wanted her parents, her mom, to meet the boys as well.” (ED)
Yes	33 (83)	9 (25)		
No	7 (17)	27 (75)		
Met in past year				“She visited us maybe 6 wk ago for 2 or 3 d and her daughter has spent a couple of weeks with us in the summer twice, and her and her husband have come another time.” (SU)
Yes	21 (53)	2 (6)	19.79, *P*<.01	
1–2 times	15 (71)	0		
3+	6 (29)	2 (100)		
No	19 (47)	34 (94)		
Contact maintenance			23.25, *P*<.01	“We have only minimal contact with the egg donor, so I send them an e-mail maybe once or twice a year with some pictures, just to stay in contact…” (ED)
Contact^a^	34 (85)	11 (31)		
Facebook	21(62)	6 (55)		
Email	14 (41)	3 (27)		
Cards/gifts/flowers	11 32)	2 (18)		
Text message	10 (29)	1 (9)		
Phone	9 (26)	0		
Skype	3 (9)	0		
No contact	6 (15)	25 (69)		
Met family			19.93, *P*<.01	“…we had a ceremony for a Jewish tradition, a Rabbi blesses the baby and gives [the baby] a Hebrew name and we paid and flew her and her sons to come up for that.” (SU)
Yes	27 (68)	6 (17)		
Siblings, parents, etc.	18 (66)	6 (100)		
Weddings	4 (15)			
Baby showers	5 (19)			
No	13 (32)	30 (83)		
Happy with level of contact				“I would be fine if it was more but I'm not bothered that it is what it is. I would not want there to be less. As I say it's just so marvelous when we do get together, it's just terrific, she's just wonderful and her husband is great. We're big fans.” (SU)
No contact	6 (15)	25 (69)		
Contact	34 (85)	11 (31)		
Content	11 (32)	3 (27)	4.17, *P*<.05	
Neutral	21 (62)	4 (36)		
Discontent	2 (6)	4 (36)		
Quality of relationship with parent A				“We have a great relationship, she is like a relative to us. …We're very close to her husband. It was a real bonding experience for us, I think they're probably some of the closest people to us really.” (positive relationship SU)
No relationship	6 (15)	25 (69)		
Relationship	34 (85)	11 (31)		
Positive	18 (53)	6 (55)	.03, *P*=.57	
Neutral	15 (44)	5 (45)		
Negative	1 (3)	0		
Quality of relationship with child				“Like when anyone comes over to the house, who's someone special, [child] gets along with all of them, like a special visitor. She and her husband are our special visitors, he doesn't react to her any differently than he does with other special visitors that we have.” (neutral, SU)
Not seen child after birth	7 (17)	27 (75)		
Met child	33 (83)	9 (25)		
Nonexistent	7 (21)	5 (56)		
Close	7 (21)	2 (22)	.04, *P*=.85	
Neutral	10 (31)	0		
Distant	8 (24)	1 (11)		
Missing	1 (3)	1 (11)		

a Some fathers engaged in multiple methods of contact maintenance, thus percentages do not equal 100.

**Table 2:** The disclosure process.

Process	n (%)	Illustrative quotes
Started the process of disclosure	33 (83)	“We've always talked about [surrogate] and who she was. So there was never a start time. It just was always been part of ongoing conversation.”
Stages of disclosure		
Two dads need help to have a baby	25 (76)	“We explained how you know, there's a nice lady. Daddy and Papa can't have kids and there's a nice lady who helped them- helped us do it. And, actually two nice ladies.”
Babies carried in women's bellies/tummies	25 (76)	“He knows that two men can't have a baby without help, only a lady can have a baby and he grew up in [surrogate]’s tummy and he was born to her but he was our baby and we loved him always.”
Specific reference to the surrogate	23 (70)	“What we said is like that they were in [surrogate]. The last time she was pregnant and I made that a point. I told all of them that they had been in there as well. That's how babies are created.”
Disclosure of the donated egg (n = 36)	12 (36)	“We haven't gone into the genetics of there being an egg donor separate from [surrogate]. He hasn't asked about it and I feel like at this stage the whole idea of genetics seems complicated, but I don't feel we're keeping it a secret, I feel we're waiting until he's older.”
Disclosure of whose sperm was used	7 (21)	“It hasn't come up that genetically, biologically they're both mine. We haven't talked about that at all yet and I think I want to make that story clear and at some point I'm going to start getting some questions and we'll kind of round the gaps from there.”
Materials		
Children's books about families/reproduction	14 (42)	“We have books about India. They are Indian. I feel like I need to celebrate their heritage, which we know nothing about.”
Photos of the surrogate	9 (27)	“We've always spoken of the surrogate as being a very important person, in the role, we've had a picture of her, we moved here about a year ago and in our old apartment we used to have like dozens of pictures up and one of them would be of the surrogate.”
Homemade books/photo albums/videos	5 (15)	“We show them the birth book, you know, of the day they were born, and there are pictures of [surrogate] and her family in it.”
Children's understanding		
None	3 (8)	“She understands the surrogate part, but not the egg donor bit. She knows that she came out of [surrogate] and she knows [surrogate] has other children and that's probably about it (some understanding).”
Some understanding	20 (50)	
Understands all	8 (20)	
Don't know	7 (17)	
Missing	2 (5)	

## References

[1] Gates GJ. Marriage and family: LGBT individuals and same-sex couples. Available at: www.princeton.edu/futureofchildren/publications/docs/MarriageandFamily.pdf. Accessed May 1, 2016.

[2] US Supreme Court. Obergefell v. Hodges, no. 14-556. 2015. Available at: https://supreme.justia.com/cases/federal/us/576/14-556/dissent7.html. Accessed May 1, 2016.

[3] Ethics Committee of the American Society for Reproductive Medicine. Access to fertility treatment by gays, lesbians, and unmarried persons: a committee opinion. Fertil Steril 2013;100:1524–1527. 10.1016/j.fertnstert.2013.08.042 24094420

[4] Perkins K.M., Boulet S.L., Jamieson D.J., Kissin D.M. (2016). National Assisted Reproductive Technology Surveillance System (NASS) Group. Trends and outcomes of gestational surrogacy in the United States. Fertil Steril.

[5] Golombok S. (2015). Modern families: parents and children in new family forms.

[6] Jadva V., Blake L., Casey P., Golombok S. (2012). Surrogacy families 10 years on: relationship with the surrogate, decisions over disclosure and children’s understanding of their surrogacy origins. Hum Reprod.

[7] MacCallum F., Lycett E., Murray C., Jadva V., Golombok S. (2003). Surrogacy: the experience of commissioning couples. Hum Reprod.

[8] Carone N, Baiocco R, Lingiardi V. “When the womb is overseas”: experiences of transnational surrogacy and relationship with the surrogate during and after the birth in gay father families. Reprod Biomed Online. In press. 10.1016/j.rbmo.2016.10.010 27839744

[9] Smietana M., Jennings S., Herbrand C., Golombok S., Freeman T., Graham S., Ebtehaj F., Richards M. (2014). Family relationships in gay father families with young children in Belgium, Spain and the United Kingdom. Relatedness in assisted reproduction. Families, origins and identities.

[10] Greenfeld D., Seli E. (2011). Gay men choosing parenthood through assisted reproduction: medical and psychosocial considerations. Fertil Steril.

[11] Ziv I., Freund-Eschar Y. (2015). The pregnancy experience of gay couples expecting a child through overseas surrogacy. Fam J.

[12] Pande A. (2010). Commercial surrogacy in India: manufacturing a perfect mother-worker. Signs.

[13] Harper J.C., Kennett D., Reisel D. (2016). The end of donor anonymity: how genetic testing is likely to drive anonymous gamete donation out of business. Hum Reprod.

[14] Freeman T., Freeman T., Graham S., Ebtehaj F., Richards M. (2014). Introduction. Relatedness in assisted reproduction: families, origins and identities.

[15] Ehrensaft D. (2005). Mommies, daddies, donors, surrogates: answering though questions and building strong families.

[16] Mitchell V., Green R.J. (2007). Different storks for different folks: gay and lesbian parents’ experiences with alternative insemination and surrogacy. J GLBT Fam Stud.

[17] Blake L., Zadeh S., Statham H., Freeman T., Freeman T., Graham S., Ebtehaj F., Richards M. (2014). Families created by assisted reproduction: children’s perspectives. Relatedness in assisted reproduction: families, origins and identities.

[18] Deakin H., Wakefield K. (2014). Skype interviewing: reflections of two PhD researchers. Qual Res.

[19] Quinton D., Rutter M. (1988). Parenting breakdown: the making and breaking of inter-generational links.

[20] Krippendorf K. (2013). Content analysis: an introduction to its methodology.

[21] Sandelowski M. (2000). Focus on research methods: whatever happened to qualitative description?. Res Nurs Health.

[22] Sandelowski M. (2010). What’s in a name? Qualitative description revisited. Res Nurs Health.

[23] Jadva V., Murray C., Lycett E., MacCallum F., Golombok S. (2003). Surrogacy: the experiences of surrogate mothers. Hum Reprod.

[24] Jadva V., Imrie S., Golombok S. (2015). Surrogate mothers 10 years on: a longitudinal study of psychological wellbeing and relationships with the parents and child. Hum Reprod.

[25] Blyth E., Kramer W., Schnieder J. (2013). Perspectives, experiences, and choices of parents of children conceived following oocyte donation. Reprod BioMed.

[26] Murphy D.A. (2015). Gay men pursuing parenthood through surrogacy: reconfiguring kinship.

[27] Dempsey D. (2013). Surrogacy, gay male couples and the significance of biogenetic paternity. New Genet Soc.

[28] Berkowitz D., Marsiglio W. (2007). Gay men: negotiating procreative, father, and family identities. J Marriage Fam.

[29] Brazier M., Campbell A., Golombok S. (1998). Review for Health Ministers of current arrangements for payments and regulation.

